# Lower relapse incidence with HAPLO versus MSD or MUD HCTs for AML patients with KMT2A rearrangement: a study from the Global Committee and the ALWP of the EBMT

**DOI:** 10.1038/s41408-024-01072-0

**Published:** 2024-05-27

**Authors:** Yishan Ye, Myriam Labopin, Jia Chen, Depei Wu, Tobias Gedde-Dahl, Didier Blaise, Gérard Socie, Edouard Forcade, Urpu Salmenniemi, Sébastien Maury, Jurjen Versluis, Ali Bazarbachi, Arnon Nagler, Eolia Brissot, Lin Li, Yi Luo, Jimin Shi, Fabio Ciceri, He Huang, Mohamad Mohty, Norbert Claude Gorin

**Affiliations:** 1 The First Affiliated Hospital, Zhejiang University School of Medicine, Hangzhou, China; 2 EBMT Paris Office, Hôpital Saint Antoine 184, rue du faubourg Saint Antoine, 75571 Paris, cedex 12 France; 3 First Affiliated Hospital of Soochow University, Suzhou, China; 4 Oslo University Hospital, Rikshospitalet, Oslo, Norway; 5 Program of Transplant and cellular immunotherapy, Department of Hematology, Institut Paoli Calmettes, Management Sport Cancer (MSC) Lab, Aix Marseille University (AMU), Marseille, France; 6 Saint-Louis Hospital, BMT Unit, Paris, France; 7 CHU Bordeaux, Hopital Haut-Leveque, Pessac, France; 8 HUCH Comprehensive Cancer Center, Helsinki, Finland; 9 Hôpital Henri Mondor, Creteil, France; 10 Erasmus MC Cancer Institute, Rotterdam, Netherlands; 11 Bone Marrow Transplantation Program, Department of Internal Medicine, American University of Beirut Medical Center, Beirut, Lebanon; 12 Department of Bone Marrow Transplantation, Chaim Sheba Medical Center, Tel-Hashomer, Israel; 13 Department of Hematology and Cell therapy, Hospital Saint-Antoine, Sorbonne University, Paris, France; 14 Ospedale San Raffaele s.r.l., Haematology and BMT, Milano, Italy

**Keywords:** Acute myeloid leukaemia, Stem-cell research

To the Editor,

KMT2A-rearranged (KMT2Ar) AML has been categorized as adverse-risk with the exception of t(9;11)(p21.3;q23.3)/KMT2A-MLLT3 considered as intermediate-risk in the 2022 European LeukemiaNet (ELN) classification [1]. Pigneux et al. reported on 159 adult patients with KMT2Ar AML allografted in remission first (CR1) or second (CR2) between 2000 and 2010. The 2-year overall survival (OS) and leukemia-free survival (LFS) were 56 ± 4% and 51 ± 4%, respectively, indicating the potential role of allo-HCT in significantly improving the prognosis for adult patients with KMT2Ar AML [2]. Different KMT2A fusion partners have been observed to predict survival in pediatric KMT2Ar AML [3, 4], but their prognostic value in the transplant setting remains unclear. Since haploidentical donor HCTs (Haplo-HCT) might exert superior graft-versus-leukemia effects than Matched Sibling and Matched Unrelated Donor (MSD/MUD) transplants, especially in high-risk AML [5, 6], we sought to discover if Haplo-HCT could bring benefit to AML patients with adverse-risk KMT2Ar (t(9;11) excluded) through relapse prevention. We also wished to investigate the prognostic value of different KMT2A fusion partners.

We therefore conducted a retrospective international multi-center study using the EBMT (European Society for Blood and Marrow Transplantation) registry. The study protocol was approved by the EBMT and the institutional review board of each site. All patients had provided informed consent for data collection before transplantation. The study was conducted as per the Declaration of Helsinki and Good Clinical Practice guidelines.

The eligibility criteria were as follows: (1) adult patients ≥18 years old; (2) first allo-HCT between January 2010 and December 2022; (3) eligible diagnosis: KMT2Ar AML in CR1 with available data on translocation involving 11q23 in the registry; (4) allo-HCTs from either a matched sibling (MSD), a 10/10 MUD or a T cell replete Haplo-HCT. The exclusion criteria were as follows: (1) allo-HCTs from mismatched UD (<10/10), umbilical cord blood, grafts with ex-vivo manipulation, or previous allo-HCT; (2) KMT2Ar AML with t(9;11); (3) patients without details on 11q23 chromosomal involvement. The conditioning regimens were defined as myeloablative conditioning (MAC) or reduced-intensity conditioning (RIC) according to the established definitions [7]. Neutrophil and platelet engraftment was defined as absolute neutrophil count exceeding 0.5 × 10^9^/L and platelet count exceeding 20 × 10^9^/L for 3 consecutive days without transfusion, respectively.

The primary study endpoint was LFS. Secondary endpoints were engraftment, OS, RI, non-relapse mortality (NRM), grade II-IV and grade III-IV acute graft-versus-host disease (GVHD) (aGVHD), chronic GVHD (cGVHD) and extensive cGVHD, and GVHD-free, relapse-free survival (GRFS). All endpoints were measured from the date of transplantation. The follow-up time was calculated using the reverse Kaplan–Meier method.

OS was defined as the time from transplant to death from any cause. LFS was defined as survival with no evidence of relapse or progression. GRFS events were defined as the first event among grade III–IV aGVHD, extensive cGVHD, relapse, and death from any cause. Acute and cGVHD were diagnosed and graded using the revised Glucksberg [8] and the NIH criteria [9], respectively. Patients’ characteristics were compared between the different donor types using the Kruskal–Wallis test for continuous variables and the chi-squared or Fisher’s exact test for categorical variables. The probabilities of OS, LFS, and GRFS were calculated using the Kaplan-Meier estimate. The probabilities of RI, NRM, aGVHD, and cGVHD were estimated using cumulative incidence curves. Both relapse and death were competing risks for GVHD. Univariate analyses were performed using the log-rank test for LFS, OS, and GRFS, and Gray’s test for cumulative incidence estimates. Multivariate analysis was performed using a Cox proportional-hazards regression model, which included factors differing in distribution between the groups (with a *p* value less than 0.10), factors known to be associated with outcomes, plus a center frailty effect to take into account the heterogeneity across centers. GVHD prevention was not included in the Cox regression as it was related to donor type. Results were expressed as the hazard ratio (HR) with a 95% confidence interval (95% CI). All tests were two-sided with a type 1 error rate fixed at 0.05. Statistical analyses were performed with SPSS 25.0 (IBM Corp., Armonk, NY, USA) and R 4.0.2 (R Core Team (2020. R: A language and environment for statistical computing. R Foundation for Statistical Computing, Vienna, Austria. URL https://www.R-project.org/).

Data from 586 adult AML patients with adverse-risk KMT2A rearrangement receiving a first allo-HCT in CR1 in 183 EBMT centers between January 2010 and December 2022 were analyzed. Patients received an allo-HCT from 201 MSD, 256 MUD, and 129 Haplo, respectively. Translocation subsets t(6;11), t(11;19), t(10;11), or ‘other’ translocations (listed in Supplementary Table S1) accounted for 27.8%, 37.5%, 23.9%, and 10.8%, respectively. Table 1 describes the patient, disease, and transplant characteristics of the three cohorts. Details of conditioning regimens are summarized in Supplementary Table S2, and regimens for GVHD prevention are summarized in Supplementary Table S3. Post-transplant cyclophosphamide (PTCy) was more frequently used in Haplo-HCT (57.8%) than in MSD (8%) or MUD (7.1%) to prevent GVHD. In vivo T-cell depletion was more commonly used in MUD (81.2%), followed by MSD (46.3%) and Haplo-HCT (39.8%). Engraftment rates were comparable among the three cohorts (MSD 99.5%, MUD 98.4%, and Haplo 96.9%).

**Table 1:** Patient, disease, and transplant characteristics.

	Overall (*n* = 586)	MSD (*n* = 201)	MUD 10/10 (*n* = 256)	Haplo (*n* = 129)	*P*
Follow-up: months, median [IQR]	36.0 [31.1–40.5]	42.0 [35.4–48.1]	35.8 [24.8–44.8]	30.0 [23.9–36.0]	0.009
Patient age: years, median [IQR]	44.9 [32.3–56.6]	46.8 [32.5–55.6]	45.9 [32.6–58.6]	40.5 [30.2–52.3]	0.027
Year of transplant: median (range)	2018 (2010–2022)	2018 (2010–2022)	2018 (2010–2022)	2018 (2010–2022)	0.0002
Time diagnosis to HCT, months, median [IQR]	4.3 [1.7–19.5]	4 [3.4–5]	4.7 [3.6–5.6]	4.5 [3.6–5.7]	0.0009
Type of AML, *n* (%)					
De novo	517 (88.2%)	177 (88.1%)	224 (87.5%)	116 (89.9%)	0.78
Secondary	69 (11.8%)	24 (11.9%)	32 (12.5%)	13 (10.1%)	
*Translocation involving 11q23, n (%)*					
t (6;11)	163 (27.8%)	62 (30.8%)	57 (22.2%)	44 (34.1%)	0.18
t (11;19)	220 (37.5%)	69 (34.3%)	105 (41%)	46 (35.6%)	
t (10;11)	140 (23.9%)	47 (23.4%)	68 (26.6%)	25 (19.4%)	
Other translocations^a^	63 (10.8%)	23 (11.5%)	26 (10.2%)	14 (10.9%)	
*Monosomal karyotype (MK), n (%)*					0.29
Not MK	489 (88.1%)	169 (88%)	215 (86.3%)	105 (92.1%)	
MK	66 (11.9%)	23 (12%)	34 (13.7%)	9 (7.9%)	
missing	31	9	7	15	
*Complex karyotype (CK), n (%)*					0.45
Not CK	406 (72.6%)	131 (67.5%)	187 (75.1%)	88 (75.9%)	
CK	153 (27.4%)	63 (32.5%)	62 (24.9%)	28 (24.1%)	
missing	27	7	7	13	
*Patient sex, n (%)*					0.82
Male	296 (50.7%)	103 (51.8%)	126 (49.2%)	67 (51.9%)	
Female	288 (49.3%)	96 (48.2%)	130 (50.8%)	62 (48.1%)	
missing	2	2	0	0	
*Donor sex, n (%)*					0.0002
Male	371 (63.6%)	108 (54%)	184 (72.4%)	79 (61.2%)	
Female	212 (36.4%)	92 (46%)	70 (27.6%)	50 (38.8%)	
missing	3	1	2	0	
*Female to male combination, n (%)*					0.002
No	488 (83.7%)	155 (77.9%)	229 (89.8%)	104 (80.6%)	
Yes	95 (16.3%)	44 (22.1%)	26 (10.2%)	25 (19.4%)	
missing	3	2	1	0	
*Cell source, n (%)*					N/A
BM	66 (11.3%)	22 (10.9%)	25 (9.8%)	19 (14.7%)	
PB	488 (83.3%)	175 (87.1%)	231 (90.2%)	83 (64.3%)	
BM + PB	12 (2%)	4 (2%)		8 (6.2%)	
BM + CB	1 (0.2%)			1 (0.8%)	
PB + CB	7 (1.2%)			7 (5.4%)	
BM + PB + CB	11 (2%)			11 (8.6%)	
*Karnofsky score, n (%)*					0.001
<90	147 (27.4%)	46 (24.2%)	58 (23.9%)	43 (41.7%)	
≥90	389 (72.6%)	144 (75.8%)	185 (76.1%)	60 (58.3%)	
missing	50	11	13	26	
*Conditioning, n (%)*					0.006
MAC	342 (60.2%)	106 (53%)	154 (60.9%)	82 (71.3%)	
RIC	226 (39.8%)	94 (47%)	99 (39.1%)	33 (28.7%)	
missing	18	1	3	14	
*Patient CMV, n (%)*					0.003
negative	219 (38.8%)	59 (29.6%)	106 (41.9%)	54 (47.8%)	
positive	346 (61.2%)	140 (70.4%)	147 (58.1%)	59 (52.2%)	
missing	21	2	3	16	
*Donor CMV, n (%)*					0.016
negative	263 (47%)	77 (39.1%)	133 (52.6%)	53 (48.6%)	
positive	296 (53%)	120 (60.9%)	120 (47.4%)	56 (51.4%)	
missing	27	4	3	20	
*PTCy, n (%)*					<0.0001
No PTCy	471 (81.3%)	183 (92%)	234 (92.9%)	54 (42.2%)	
PTCy	108 (18.7%)	16 (8%)	18 (7.1%)	74 (57.8%)	
missing	7	2	4	1	
*In vivo T-cell depletion (TCD), n (%)*					<0.0001
No in vivo TCD	233 (39.9%)	108 (53.7%)	48 (18.8%)	77 (60.2%)	
In vivo TCD	351 (60.1%)	93 (46.3%)	207 (81.2%)	51 (39.8%)	
missing	2	0	1	1	
*Engraftment after HCT, n (%)*					0.17
Graft failure	9 (1.6%)	1 (0.5%)	4 (1.6%)	4 (3.1%)	
Engrafted	569 (98.4%)	196 (99.5%)	250 (98.4%)	123 (96.9%)	
Missing	8	4	2	2	
*Acute GVHD, n (%)*					N/A
Grade I	94 (16.3%)	21 (10.7%)	46 (18.2%)	27 (21.3%)	
Grade II	71 (12.3%)	19 (9.7%)	38 (15%)	14 (11%)	
Grade III	41 (7.1%)	8 (4.1%)	22 (8.7%)	11 (8.7%)	
Grade IV	35 (6.1%)	9 (4.6%)	18 (7.1%)	8 (6.3%)	
Present, grade unknown	8 (1.4%)	4 (2%)	3 (1.2%)	1 (0.8%)	
No aGVHD present (Grade 0)	327 (56.8%)	135 (68.9%)	126 (49.8%)	66 (52%)	
missing	10	5	3	2	

Abbreviations: *AML* acute myeloid leukemia, *MSD* matched sibling donor, *MUD* matched unrelated donor, *Haplo* haploidentical donor, *IQR* interquartile range, *HCT* hematopoietic cell transplantation, *BM* bone marrow, *PB* peripheral blood, *CB* cord blood, *MAC* myeloablative conditioning, *RIC* reduced-intensity conditioning, *CMV* cytomegalovirus, *PTCy* post-transplant cyclophosphamide, *GVHD* graft-versus-host disease;

^a^Other translocations listed in Supplementary Table S1.

The 2-year and 5-year cumulative RI, NRM, OS, LFS, and GRFS, as well as the acute and cGVHD incidences of the entire cohort, are shown in Fig. 1 and Supplementary Table S4. Supplementary Table S5 shows the results of univariate analyses. The 2-year and 5-year cumulative RIs were 34.9% (95% CI: 30.9–37.1) and 40.8% (95% CI: 36.1–45.4), respectively. Meanwhile, the 2-year and 5-year incidences of NRM were 15.1% (95% CI: 12.1–18.4) and 16.9% (95% CI: 13.7–20.4), respectively. Finally, the 2-year and 5-year OS were 60.8% (95% CI: 56.2–65) and 47.8% (95% CI: 42.6–52.8), and the 2-year and 5-year LFS were 50% (95% CI: 45.5–54.4) and 42.3% (95% CI: 37.5–47), respectively.

**Fig. 1:**
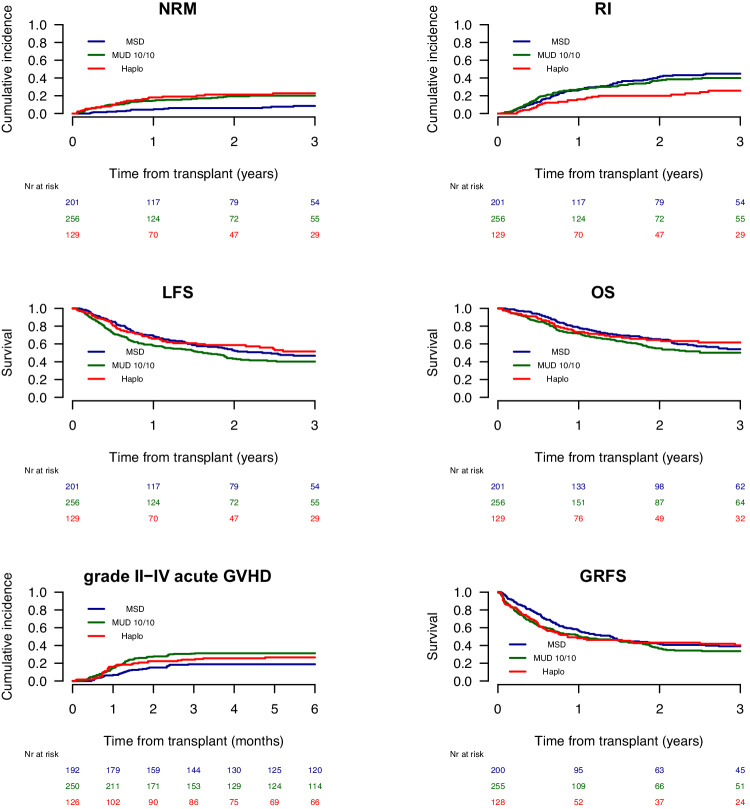
Transplant outcomes of the HAPLO, MSD and MUD cohorts for adverse-risk KMT2Ar AML. NRM, RI, LFS, OS, GRFS, and grade II–IV acute GVHD of the HAPLO, MSD, and MUD cohorts.

Table 2 shows the results of multivariate analyses: Haplo-HCT was associated with a lower RI (HR = 0.46, 95% CI: 0.28–0.77; *p* < 0.01) but a higher incidence of NRM (HR = 2.36, 95% CI: 1.17–4.78; *p* < 0.01) compared with MSD recipients, with no significant impact on LFS, OS or GRFS. Importantly, when compared with MUD, Haplo-HCT was associated with a significantly lower risk of RI (HR = 0.43, 95% CI: 0.25–0.72; *p* < 0.01) and similar NRM, which translated into better LFS (HR = 0.55, 95% CI: 0.37–0.82; *p* < 0.01), OS (HR = 0.62, 95% CI: 0.4–0.95; *p* = 0.03) and GRFS (HR = 0.71, 95% CI: 0.51–0.98; *p* = 0.04). Concerning translocation type, t(11;19) was associated with a lower RI (HR = 0.64, 95% CI: 0.43–0.94), a better LFS (HR = 0.66, 95% CI: 0.48–0.91), OS (HR = 0.63, 95% CI: 0.45–0.89) and GRFS (HR = 0.75, 95% CI: 0.57–1) compared with t(6;11). Translocations other than t(6;11), t(11;19), or t(10;11) were associated with a lower RI (HR = 0.45, 95% CI: 0.24–0.87) and a better OS (HR = 0.57, 95% CI: 0.34–0.95) compared with t(6;11).

**Table 2:** Multivariate analysis of factors affecting allo-HCT outcomes for adverse-risk KMT2Ar AML patients.

	2-year results									
	Relapse		NRM		LFS		OS		GRFS	
	HR (95% CI)	*P* value	HR (95% CI)	*P* value	HR (95% CI)	*P* value	HR (95% CI)	*P* value	HR (95% CI)	*P* value
MSD (ref)	1		1		1		1		1	
MUD 10/10	1.08 (0.77–1.51)	0.65	2.73 (1.51–4.94)	0.0009	1.37 (1.03–1.83)	0.032	1.21 (0.89–1.64)	0.21	1.22 (0.95–1.58)	0.13
Haplo	0.46 (0.28–0.77)	0.003	2.36 (1.17–4.78)	0.017	0.76 (0.51–1.13)	0.17	0.75 (0.48–1.16)	0.19	0.86 (0.61–1.21)	0.39
Haplo vs MUD	0.43 (0.25–0.72)	0.001	0.86 (0.48–1.57)	0.63	0.55 (0.37–0.82)	0.003	0.62 (0.4–0.95)	0.027	0.71 (0.51–0.98)	0.037
t (6;11) (ref)	1		1		1		1		1	
t (11;19)	0.64 (0.43–0.94)	0.022	0.7 (0.4–1.24)	0.22	0.66 (0.48–0.91)	0.012	0.63 (0.45–0.89)	0.008	0.75 (0.57–1)	0.049
t (10;11)	0.84 (0.57–1.25)	0.4	0.48 (0.23–1.02)	0.055	0.76 (0.54–1.08)	0.12	0.73 (0.5–1.05)	0.087	0.75 (0.55–1.02)	0.068
Other translocations^a^	0.45 (0.24–0.87)	0.016	1.15 (0.58–2.29)	0.68	0.68 (0.43–1.08)	0.11	0.57 (0.34–0.95)	0.033	0.66 (0.43–1.01)	0.056
age (per 10 y)	1.01 (0.89–1.14)	0.92	1.19 (0.98–1.45)	0.075	1.06 (0.95–1.17)	0.29	1.09 (0.97–1.22)	0.13	1.04 (0.95–1.14)	0.43
Year of HCT	1.06 (1–1.12)	0.034	0.96 (0.9–1.04)	0.34	1.03 (0.98–1.07)	0.26	1.01 (0.96–1.06)	0.68	1.05 (1.01–1.09)	0.016
CK	1.24 (0.88–1.74)	0.23	0.81 (0.46–1.45)	0.48	1.09 (0.82–1.47)	0.55	1.15 (0.84–1.57)	0.38	0.98 (0.76–1.28)	0.9
MK	1.59 (1.04–2.45)	0.033	1.02 (0.45–2.28)	0.97	1.47 (1.01–2.14)	0.046	1.36 (0.9–2.05)	0.14	1.26 (0.89–1.78)	0.19
RIC vs MAC	0.9 (0.64–1.27)	0.54	0.91 (0.54–1.54)	0.72	0.89 (0.67–1.2)	0.45	0.97 (0.71–1.32)	0.83	0.88 (0.68–1.14)	0.34
Female to Male	0.9 (0.6–1.36)	0.62	1.2 (0.66–2.2)	0.55	0.97 (0.69–1.37)	0.88	1 (0.69–1.44)	0.99	0.98 (0.72–1.32)	0.87
Patient CMV positive	0.82 (0.58–1.15)	0.25	1.09 (0.64–1.87)	0.74	0.87 (0.65–1.17)	0.36	0.91 (0.67–1.25)	0.57	0.91 (0.7–1.17)	0.44
Donor CMV positive	1.17 (0.84–1.62)	0.36	1.02 (0.62–1.68)	0.93	1.1 (0.83–1.45)	0.5	1 (0.75–1.35)	0.98	1.02 (0.8–1.3)	0.87

	180-day results				2-year results			
	aGVHD II-IV		aGVHD III-IV		cGVHD		Extensive cGVHD	
	HR (95% CI)	P value	HR (95% CI)	P value	HR (95% CI)	P value	HR (95% CI)	P value
MSD (ref)	1		1		1		1	
MUD 10/10	1.82 (1.19–2.78)	0.005	1.78 (0.96–3.32)	0.069	0.93 (0.65–1.33)	0.69	1.09 (0.64–1.85)	0.76
Haplo	1.36 (0.79–2.35)	0.27	1.21 (0.54–2.72)	0.64	0.89 (0.57–1.4)	0.63	1.29 (0.7–2.4)	0.42
Haplo vs MUD	0.75 (0.45–1.23)	0.25	0.68 (0.33–1.38)	0.29	0.96 (0.61–1.52)	0.864	1.19 (0.64–2.21)	0.58
t (6;11) (ref)	1		1		1		1	
t (11;19)	0.96 (0.61–1.49)	0.84	1.29 (0.64–2.62)	0.48	0.82 (0.55–1.2)	0.3	0.93 (0.54–1.61)	0.8
t (10;11)	0.93 (0.57–1.52)	0.78	1.47 (0.7–3.11)	0.31	0.67 (0.43–1.04)	0.074	0.6 (0.31–1.17)	0.14
Other translocations^a^	0.71 (0.36–1.41)	0.33	1.32 (0.51–3.44)	0.56	0.67 (0.38–1.18)	0.16	0.6 (0.25–1.45)	0.26
age (per 10 y)	0.95 (0.83–1.09)	0.48	1.03 (0.84–1.26)	0.77	1.03 (0.91–1.17)	0.62	1.02 (0.84–1.23)	0.86
Year of HCT	0.98 (0.93–1.04)	0.59	1.08 (0.99–1.17)	0.092	1 (0.94–1.05)	0.9	1.04 (0.96–1.13)	0.32
CK	0.91 (0.6–1.4)	0.68	0.84 (0.45–1.57)	0.59	0.83 (0.57–1.21)	0.34	1.03 (0.6–1.77)	0.91
MK	0.8 (0.43–1.47)	0.47	0.72 (0.3–1.75)	0.47	1.2 (0.72–2)	0.49	0.56 (0.22–1.44)	0.23
RIC vs MAC	1.04 (0.7–1.56)	0.85	0.99 (0.56–1.77)	0.98	0.76 (0.53–1.09)	0.13	0.81 (0.48–1.38)	0.44
Female to Male	1.18 (0.74–1.88)	0.48	0.95 (0.46–1.96)	0.89	1.59 (1.09–2.33)	0.016	1.44 (0.83–2.5)	0.19
Patient CMV positive	0.97 (0.65–1.45)	0.87	1.12 (0.63–2)	0.69	0.79 (0.54–1.15)	0.21	1.12 (0.65–1.91)	0.69
Donor CMV positive	0.76 (0.52–1.12)	0.16	0.65 (0.38–1.14)	0.13	0.88 (0.62–1.24)	0.47	0.88 (0.54–1.44)	0.62

Abbreviations: *AML* acute myeloid leukemia, *NRM* non-relapse mortality, *LFS* leukemia-free survival, *OS* overall survival, *GRFS* GVHD-free relapse-free survival, *GVHD* graft-versus-host disease, *aGVHD* acute GVHD, *cGVHD* chronic GVHD, *HR* hazard ratio, *95% CI* 95% confidence interval, *MSD* matched sibling donor, *MUD* matched unrelated donor, *Haplo* haploidentical donor, *HCT* hematopoietic cell transplantation, *CK* complex karyotype, *MK* monosomal karyotype, *MAC* myeloablative conditioning, *RIC* reduced-intensity conditioning, *CMV* cytomegalovirus.

^a^Other translocations listed in Supplementary Table S1.

A total of 237 (40%) patients died during the study period (Supplementary Table S6). The original disease was the most common cause of death in the MSD (70.6%), MUD (56.1%), and Haplo-HCT (39%) groups. Proportions of GVHD-related deaths were 23.4% in MUD, 17.1% in Haplo, and 8.2% in the MSD group. The proportion of deaths due to infection was 29.3%, 13.1%, and 9.4% in the Haplo, MUD, and MSD groups, respectively.

The present analysis constitutes the largest global series and provides the first comprehensive comparison of different donor stem cell sources in a homogeneous population of adverse-risk KMT2Ar AML patients excluding t(9;11), all transplanted in CR1. The major finding of this study is a significantly decreased RI in patients who underwent Haplo-HCT compared with MSD- or MUD-HCTs. A higher than expected NRM contributed to the absence of statistically significant differences in LFS between the 3 groups (2-year LFS, HAPLO 58.7%, MSD 52.8%, MUD 43.4%). When compared with MUD patients, however, NRM similar to Haplo-HCT, Haplo-HCT resulted in significantly better survival outcomes.

The question of the prognostic significance of the KMT2Ar fusion partners has remained uncertain [3]. Data from the current study support the prognostic value of t(6;11) and t(10;11) in predicting poor outcomes and indicate that t(11;19) might be associated with good prognosis in the transplant setting. It should be noted that the heterogeneity of KMT2Ar AML is not only derived from different fusion partners but also results from many additional factors such as the cell of origin [10, 11], co-mutations [12], or EVI1 expression level [11], which in turn also modify the prognosis of these patients.

This study has the limitations of retrospective registry studies. It was designed to compare worldwide in a high-risk AML patient population the impact of three different donor categories, MSD, MUD, and Haplo, and not other specific factors of importance, such as the nature of the pretransplant regimen or GVH prophylaxis. For each transplant, each team selected what it believed to be the best approach consisting, among others, of the best available donor, the most appropriate conditioning regimen, and its own GVH prophylaxis. In particular a comparison of PTCY versus no-PTCY regimen for GVH prophylaxis would not be feasible. Obviously and unfortunately, a prospective randomized study testing the donor choice for allotransplant in adverse KMT2Ar AML would be impossible to conduct. This study supports the use of haploidentical donors for the transplantation of KMT2Ar AML patients. Efforts to reduce NRM and combined treatment with a menin inhibitor before transplant or using it as part of maintenance therapy might further improve the transplant outcome.

### Supplementary information

Supplementary materials

## Data Availability

The datasets generated during and/or analyzed during the current study are available from the corresponding author upon reasonable request.
