# Supplementary material for: Lower relapse incidence with HAPLO versus MSD or MUD HCTs for AML patients with KMT2A rearrangement: a study from the Global Committee and the ALWP of the EBMT
Source: Blood Cancer J. 2024 May 27;14(1):85. doi: 10.1038/s41408-024-01072-0 (PMC11130289; doi:10.1038/s41408-024-01072-0)
Supplement: Supplementary file 1 — Supplementary materials [file 41408_2024_1072_MOESM1_ESM.docx]

**Supplementary Table S1. KMT2A translocations other than t(6;11), t(11;19), t(10;11)**

| **Translocations** | **N=63** |
| --- | --- |
| t (11;17) | 25 |
| t (1;11) | 7 |
| t (4;11) | 5 |
| t (7;11) | 5 |
| t (11;22) | 4 |
| t (5;11) | 3 |
| t (11;12) | 2 |
| t (11;14) | 2 |
| t (11;16) | 2 |
| t (2;11) | 2 |
| t (X;11) | 2 |
| t (11;13) | 1 |
| t (11;15) | 1 |
| t (11;20) | 1 |
| t (3;11) | 1 |

**Supplementary Table S2. Conditioning regimens**

| **Conditioning regimen n (%)** | **Overall**  **(n=586)** | **MSD**  **(n=201)** | **MUD 10/10**  **(n=256)** | **Haplo**  **(n=129)** |
| --- | --- | --- | --- | --- |
| BuCy | 137 (23.4%) | 53 (26.4%) | 42 (16.4%) | 42 (32.6%) |
| BuFlu | 160 (27.3%) | 62 (30.8%) | 84 (32.8%) | 14 (10.9%) |
| FLAMSA-Bu | 35 (6%) | 23 (11.4%) | 12 (4.7%) | 0 (0%) |
| TBF | 81 (13.8%) | 10 (5%) | 18 (7%) | 53 (41.1%) |
| FluMel | 33 (5.6%) | 16 (8%) | 17 (6.6%) | 0 (0%) |
| FLAMSA-Mel | 2 (0.3%) | 1 (0.5%) | 0 (0%) | 1 (0.8%) |
| FTM | 4 (0.7%) | 2 (1%) | 1 (0.4%) | 1 (0.8%) |
| FluTreo | 37 (6.3%) | 7 (3.5%) | 25 (9.8%) | 5 (3.9%) |
| FLAMSA-Treo | 2 (0.3%) | 1 (0.5%) | 1 (0.4%) | 0 (0%) |
| FluCy | 1 (0.2%) | 0 (0%) | 1 (0.4%) | 0 (0%) |
| Cy-TBI | 32 (5.5%) | 9 (4.5%) | 23 (9%) | 0 (0%) |
| Flu-TBI | 41 (7%) | 11 (5.5%) | 21 (8.2%) | 9 (7%) |
| Bu-TBI | 1 (0.2%) | 0 (0%) | 0 (0%) | 1 (0.8%) |
| FLAMSA-TBI | 12 (2%) | 5 (2.5%) | 6 (2.3%) | 1 (0.8%) |
| Bu-Mel | 1 (0.2%) | 1 (0.5%) | 0 (0%) | 0 (0%) |
| Thiotepa based | 1 (0.2%) | 0 (0%) | 0 (0%) | 1 (0.8%) |
| Clofa based | 2 (0.3%) | 0 (0%) | 2 (0.8%) | 0 (0%) |
| Treo-Flu-Mel | 4 (0.7%) | 0 (0%) | 3 (1.2%) | 1 (0.8%) |

**Abbreviations:** MSD, matched sibling donor; MUD, matched unrelated donor; Haplo, haploidentical donor; Bu, busulfan; Cy, cyclophosphamide; Flu, fludarabine; FLAMSA, fludarabine/cytarabine/amsacrine based regimen; TBF, thiotepa/busulfan/fludarabine based regimen; Mel, melphalan; FTM, fludarabine/carmustine/thiotepa based regimen; Treo, treosulfan; TBI, total body irradiation; Clofa, clofarabine;

**Supplementary Table S3. Regimens for GVHD prevention**

| **GVHD prevention regimen, n (%)** | **Overall**  **(n=586)** | **MSD**  **(n=201)** | **MUD 10/10**  **(n=256)** | **Haplo**  **(n=129)** |
| --- | --- | --- | --- | --- |
| CSA | 58 (9.9%) | 31 (15.4%) | 25 (9.8%) | 2 (1.6%) |
| MTX | 4 (0.7%) | 0 (0%) | 4 (1.6%) | 0 (0%) |
| Tacro | 8 (1.4%) | 2 (1%) | 4 (1.6%) | 2 (1.6%) |
| MMF | 2 (0.3%) | 2 (1%) | 0 (0%) | 0 (0%) |
| CSA+MTX | 194 (33.2%) | 90 (44.8%) | 103 (40.4%) | 1 (0.8%) |
| MTX+Tacro | 14 (2.4%) | 3 (1.5%) | 11 (4.3%) | 0 (0%) |
| CSA+MMF | 189 (32.4%) | 57 (28.4%) | 76 (29.8%) | 56 (43.8%) |
| CSA+Tacro | 1 (0.2%) | 1 (0.5%) | 0 (0%) | 0 (0%) |
| CSA+MTX+MMF | 52 (8.9%) | 1 (0.5%) | 7 (2.7%) | 44 (34.4%) |
| MMF+Tacro | 40 (6.8%) | 6 (3%) | 16 (6.3%) | 18 (14.1%) |
| MMF+Siro | 7 (1.2%) | 1 (0.5%) | 4 (1.6%) | 2 (1.6%) |
| CSA+MMF+Tacro | 5 (0.9%) | 1 (0.5%) | 1 (0.4%) | 3 (2.3%) |
| Tacro+Siro | 3 (0.5%) | 2 (1%) | 1 (0.4%) | 0 (0%) |
| Other | 7 (1.2%) | 4 (2%) | 3 (1.2%) | 0 (0%) |
| missing | 2 | 0 | 1 | 1 |

**Abbreviations:** MSD, matched sibling donor; MUD, matched unrelated donor; Haplo, haploidentical donor; CSA, cyclosporine A; MTX, methotrexate; Tacro, tacrolimus; MMF, mycophenolate mofetil; Siro, sirolimus;

**Supplementary Table S4. Transplant outcomes of the entire cohort**

| **Survival outcomes (estimation (95% CI%))** | | | | | |
| --- | --- | --- | --- | --- | --- |
|  | **RI** | **NRM** | **OS** | **LFS** | **GRFS** |
| **2 years** | 34.9  (30.7-39.1) | 15.1  (12.1-18.4) | 60.8  (56.2-65) | 50  (45.5-54.4) | 39.9  (35.6-44.2) |
| **5 years** | 40.8  (36.1-45.4) | 16.9  (13.7-20.4) | 47.8  (42.6-52.8) | 42.3  (37.5-47) | 33.5  (29.1-38) |

| **GVHD incidence (estimation (95% CI%))** | | | | | |
| --- | --- | --- | --- | --- | --- |
| **aGVHD (180 days)** | | **cGVHD (2 years)** | | **cGVHD (5 years)** | |
| Grades II-IV | Grades III-IV | Overall | Extensive | Overall | Extensive |
| 26  (22.5-29.7) | 13  (10.4-15.9) | 39.8  (35.4-44.2) | 17.2  (13.9-20.9) | 41.5  (36.8-46) | 19.4  (15.8-23.4) |

**Supplementary Table S5. Univariate analysis on factors affecting allo-HCT outcomes for adverse-risk KMT2Ar AML patients**

|  |  | **2-year results** | | | | |
| --- | --- | --- | --- | --- | --- | --- |
|  |  | **Relapse** | **NRM** | **LFS** | **OS** | **GRFS** |
| **Type of donor, %[95% CI]** | MSD | 41.1%[33.6-48.4] | 6.1% [3.2-10.3] | 52.8% [45-60] | 65.4% [57.8-72.1] | 42% [34.5-49.3] |
|  | MUD 10/10 | 37.2%[30.7-43.8] | 19.3% [14.4-24.9] | 43.4% [36.6-50.1] | 55% [47.9-61.6] | 36.6% [30.2-43.1] |
|  | Haplo | 20% [13-28] | 21.3% [14.2-29.5] | 58.7% [48.8-67.3] | 64.5% [54.4-72.9] | 43.2% [33.9-52.2] |
|  | P value | **0.002** | **0.001** | **0.049** | 0.27 | 0.26 |
|  |  |  |  |  |  |  |
| **Translocation type, %[95% CI]** | t(6;11) | 42.2%[33.8-50.4] | 15.5%[10.2-21.9] | 42.2%[33.8-50.4] | 54.8%[46-62.8] | 31.4%[23.8-39.2] |
|  | t(11;19) | 29.9%[23.5-36.6] | 14.8%[10.2-20.3] | 55.3%[47.7-62.1] | 63%[55.5-69.7] | 42.4%[35.3-49.3] |
|  | t(10;11) | 41.3%[32.1-50.1] | 10.3%[5.7-16.4] | 48.5%[39-57.2] | 62%[52.4-70.1] | 41.7%[32.7-50.5] |
|  | other translocations | 18.7%[9.4-30.3] | 25.9%[14.8-38.5] | 55.4%[40.7-67.8] | 65.9%[50.9-77.3] | 49.7%[35.5-62.4] |
|  | P value | **0.01** | 0.13 | 0.08 | **0.031** | 0.1 |
|  |  |  |  |  |  |  |
| **Patient age,**  **%[95% CI]** | age<45y | 38.5%[32.2-44.7] | 12.1%[8.4-16.5] | 49.4%[42.8-55.6] | 63.2%[56.6-69.1] | 37.6%[31.4-43.7] |
|  | age≥45y | 31.5%[25.8-37.3] | 17.9%[13.5-22.9] | 50.6%[44.2-56.6] | 58.4%[51.9-64.3] | 42.2%[36.1-48.2] |
|  | P value | 0.1 | **0.03** | 0.93 | 0.39 | 0.54 |
|  |  |  |  |  |  |  |
| **Year of HCT,**  **%[95% CI]** | Year<2018 | 32.6%[26.7-38.6] | 18.1%[13.5-23.2] | 49.3%[42.8-55.5] | 59.9%[53.3-65.8] | 40.4%[34.2-46.6] |
|  | Year≥2018 | 36.7%[30.7-42.7] | 12.4%[8.7-16.6] | 50.9%[44.5-57] | 61.4%[54.8-67.3] | 39.4%[33.4-45.4] |
|  | P value | 0.25 | 0.07 | 0.84 | 0.88 | 0.65 |
|  |  |  |  |  |  |  |
| **Type of AML,**  **%[95% CI]** | de novo | 35.4%[30.9-40] | 15.5%[12.3-19] | 49.1%[44.2-53.8] | 60.5%[55.7-65.1] | 39.4%[34.8-44] |
|  | secondary AML | 30.9%[19.6-42.8] | 12.4%[5.7-21.7] | 56.8%[43.4-68.1] | 62.3%[48.7-73.3] | 43.6%[31-55.5] |
|  | P value | 0.42 | 0.96 | 0.44 | 0.52 | 0.5 |
|  |  |  |  |  |  |  |
| **CK, %[95% CI]** | not CK | 31.8%[26.8-36.8] | 16.8%[13.1-21] | 51.4%[45.9-56.6] | 63.5%[58.1-68.5] | 40.9%[35.7-46.1] |
|  | CK | 42.7%[34-51] | 10.9%[6.4-16.9] | 46.4%[37.6-54.8] | 53.2%[44.2-61.5] | 37.8%[29.5-46.1] |
|  | P value | **0.007** | 0.1 | 0.17 | 0.17 | 0.85 |
|  |  |  |  |  |  |  |
| **MK, %[95% CI]** | not MK | 33.8%[29.2-38.4] | 15.5%[12.2-19.1] | 50.7%[45.7-55.5] | 62.2%[57.3-66.8] | 40.3%[35.6-45] |
|  | MK | 44.6%[31.2-57.1] | 12.3%[5.3-22.4] | 43.2%[29.9-55.7] | 48%[33.8-60.8] | 37.2%[24.6-49.7] |
|  | P value | **0.009** | 0.32 | 0.08 | 0.24 | 0.45 |
|  |  |  |  |  |  |  |
| **Conditioning,**  **%[95% CI]** | MAC | 35%[29.5-40.6] | 14.7%[11-19] | 50.2%[44.3-55.9] | 62.5%[56.5-67.9] | 38.1%[32.5-43.6] |
|  | RIC | 35.1%[28.4-41.9] | 15.6%[10.9-21] | 49.3%[42-56.2] | 58.4%[51-65.1] | 41.9%[34.8-48.7] |
|  | P value | 0.68 | 0.65 | 0.86 | 0.56 | 0.19 |
|  |  |  |  |  |  |  |
| **Female to male, %[95% CI]** | no F->M | 34.8%[30.2-39.5] | 15.5%[12.2-19.1] | 49.7%[44.7-54.5] | 60.3%[55.2-65] | 39.6%[34.8-44.3] |
|  | F->M | 34.2%[24.1-44.5] | 13.7%[7.4-21.8] | 52.1%[40.9-62.2] | 62.1%[50.8-71.6] | 41.9%[31.3-52.2] |
|  | P value | 0.58 | 0.7 | 0.39 | 0.53 | 0.55 |
|  |  |  |  |  |  |  |
| **Patient CMV, %[95% CI]** | Pat. CMV neg. | 36.1%[29.1-43.2] | 14.7%[10-20.2] | 49.2%[41.6-56.4] | 60.7%[52.9-67.5] | 36.4%[29.4-43.3] |
|  | Pat. CMV pos | 34.3%[28.9-39.7] | 15.2%[11.4-19.4] | 50.5%[44.7-56.1] | 61.3%[55.4-66.6] | 41.7%[36-47.2] |
|  | P value | 0.59 | 0.83 | 0.7 | 0.7 | 0.19 |
|  |  |  |  |  |  |  |
| **Donor CMV,**  **%[95% CI]** | Don. CMV neg. | 34.1%[27.8-40.4] | 16.3%[11.8-21.4] | 49.7%[42.8-56.1] | 60.4%[53.4-66.6] | 37.9%[31.6-44.2] |
|  | Don. CMV pos | 36.1%[30.2-42.1] | 13.8%[9.9-18.3] | 50.1%[43.7-56.1] | 61.1%[54.7-66.9] | 41.1%[35-47.1] |
|  | P value | 0.53 | 0.59 | 0.88 | 0.73 | 0.48 |
|  |  |  |  |  |  |  |
| **In vivo TCD,**  **%[95% CI]** | no in vivo TCD | 36.2%[29.4-43.1] | 13.8%[9.4-18.9] | 50%[42.7-56.9] | 61.5%[54.1-68] | 36.9%[30.1-43.8] |
|  | in vivo TCD | 33.6%[28.3-39] | 16%[12.2-20.4] | 50.3%[44.5-55.9] | 60.4%[54.5-65.9] | 42.1%[36.5-47.6] |
|  | P value | 0.28 | 0.19 | 0.98 | 0.73 | 0.35 |
|  |  |  |  |  |  |  |
| **PTCy, %[95% CI]** | No PTCy | 37.2%[32.5-42] | 15.5%[12.2-19.1] | 47.3%[42.3-52.1] | 59.1%[54.1-63.8] | 38.5%[33.8-43.3] |
|  | PTCy | 19.8%[12.3-28.7] | 14.2%[7.9-22.4] | 66%[55.1-74.8] | 70.6%[59.7-79] | 48.2%[37.5-58] |
|  | P value | **0.031** | 0.6 | **0.022** | 0.06 | 0.25 |
|  |  |  | | | | |
|  |  | **180-day results** | | **2-year results** | |  |
|  |  | **aGVHD II-IV** | **aGVHD III-IV** | **cGVHD** | **ext. cGVHD** |  |
| **Type of donor, %[95% CI]** | MSD | 18.8%[13.6-24.7] | 8.4%[5-12.9] | 42.1%[34.5-49.5] | 17.5%[12-23.8] |  |
|  | MUD 10/10 | 31.3%[25.6-37.1] | 15.7%[11.5-20.5] | 37.1%[30.5-43.7] | 15.2%[10.5-20.6] |  |
|  | HAPLO | 26.5%[19.1-34.5] | 14.6%[9.1-21.5] | 41.7%[31.7-51.4] | 21.7%[13.9-30.6] |  |
|  | P value | **0.01** | 0.06 | 0.71 | 0.27 |  |
|  |  |  |  |  |  |  |
| **Translocation type, %[95% CI]** | t(6;11) | 26.1%[19.5-33.2] | 10.3%[6.2-15.7] | 46.2%[37.4-54.5] | 20.2%[13.6-27.8] |  |
|  | t(11;19) | 26.8%[21-32.9] | 13.7%[9.5-18.8] | 39.3%[32-46.6] | 19%[13.5-25.3] |  |
|  | t(10;11) | 27.5%[20.2-35.3] | 15.6%[10.1-22.3] | 34.7%[26.2-43.4] | 12.5%[7.1-19.4] |  |
|  | other translocations | 19.7%[10.8-30.5] | 11.5%[5-20.9] | 36.1%[22.6-49.7] | 14.3%[6.1-25.9] |  |
|  | P value | 0.63 | 0.57 | 0.48 | 0.3 |  |
|  |  |  |  |  |  |  |
| **Patient age,**  **%[95% CI]** | age<median | 27.9%[22.8-33.2] | 13.5%[9.8-17.7] | 43%[36.5-49.4] | 18.3%[13.5-23.8] |  |
|  | age>median | 24.1%[19.3-29.2] | 12.5%[9-16.7] | 36.8%[30.8-42.9] | 16.2%[11.8-21.2] |  |
|  | P value | 0.23 | 0.72 | 0.23 | 0.53 |  |
|  |  |  |  |  |  |  |
| **Year of HCT,**  **%[95% CI]** | Year<median | 29.3%[23.7-35.1] | 13.5%[9.6-18.1] | 39.9%[33.5-46.2] | 17.9%[13.2-23.2] |  |
|  | Year>median | 23.5%[19-28.3] | 12.6%[9.2-16.5] | 39.6%[33.4-45.7] | 16.6%[12-21.8] |  |
|  | P value | 0.15 | 0.78 | 0.86 | 0.85 |  |
|  |  |  |  |  |  |  |
| **Secondary AML, %[95% CI]** | de novo | 26.5%[22.7-30.5] | 13%[10.2-16.1] | 39.8%[35.1-44.5] | 17.4%[13.8-21.3] |  |
|  | Sec AML | 22.1%[13-32.6] | 13.2%[6.5-22.5] | 39.7%[27-52.2] | 16%[7.8-26.9] |  |
|  | P value | 0.49 | 0.92 | 0.83 | 0.82 |  |
|  |  |  |  |  |  |  |
| **CK, %[95% CI]** | not CK | 27.2%[22.9-31.7] | 13.4%[10.2-17] | 42%[36.6-47.3] | 17.9%[13.9-22.4] |  |
|  | CK | 22.8%[16.4-29.9] | 10.2%[6-15.7] | 36.3%[27.9-44.7] | 17.3%[11.1-24.6] |  |
|  | P value | 0.34 | 0.32 | 0.13 | 0.39 |  |
|  |  |  |  |  |  |  |
| **MK, %[95% CI]** | not MK | 26.8%[22.9-30.9] | 12.8%[10-16] | 40%[35.2-44.8] | 18.8%[15.1-22.9] |  |
|  | MK | 20%[11.3-30.6] | 9.2%[3.7-17.8] | 42.6%[28.2-56.2] | 8.6%[2.6-19.1] |  |
|  | P value | 0.23 | 0.43 | 0.79 | 0.08 |  |
|  |  |  |  |  |  |  |
| **Conditioning,**  **%[95% CI]** | MAC | 26.7%[22-31.6] | 13.1%[9.7-17] | 41.1%[35.3-46.8] | 18%[13.7-22.8] |  |
|  | RIC | 25.6%[20-31.6] | 12.5%[8.5-17.3] | 36.8%[29.9-43.7] | 16.4%[11.4-22.3] |  |
|  | P value | 0.75 | 0.83 | 0.33 | 0.54 |  |
|  |  |  |  |  |  |  |
| **Female to male, %[95% CI]** | no F->M | 25.9%[22-29.9] | 13.5%[10.6-16.7] | 37.1%[32.3-41.9] | 16.4%[12.8-20.4] |  |
|  | F->M | 27.4%[18.6-36.8] | 10.9%[5.6-18.3] | 54.7%[43-65] | 21.9%[13.4-31.8] |  |
|  | P value | 0.75 | 0.48 | **0.002** | 0.12 |  |
|  |  |  |  |  |  |  |
| **Patient CMV,**  **%[95% CI]** | Pat. CMV neg. | 29.8%[23.8-36.1] | 15.1%[10.7-20.3] | 40.2%[32.8-47.5] | 17.2%[11.8-23.5] |  |
|  | Pat. CMV pos | 24%[19.5-28.7] | 11.5%[8.3-15.2] | 38.8%[33.2-44.4] | 17.6%[13.4-22.3] |  |
|  | P value | 0.12 | 0.23 | 0.68 | 0.74 |  |
|  |  |  |  |  |  |  |
| **Donor CMV,**  **%[95% CI]** | Don. CMV neg. | 30.1%[24.6-35.8] | 16.4%[12.2-21.2] | 40.9%[34.2-47.4] | 18.5%[13.5-24.2] |  |
|  | Don. CMV pos | 22.9%[18.2-28] | 9.6%[6.5-13.4] | 37.9%[31.8-43.9] | 16.4%[12-21.4] |  |
|  | P value | 0.06 | 0.018 | 0.53 | 0.81 |  |
|  |  |  |  |  |  |  |
| **In vivo TCD,**  **%[95% CI]** | no in vivo TCD | 25.5%[20-31.3] | 10.7%[7.1-15.1] | 45.9%[38.5-52.9] | 22.1%[16.4-28.5] |  |
|  | in vivo TCD | 26.2%[21.6-31] | 14.6%[11.1-18.6] | 35.8%[30.2-41.4] | 14%[10.2-18.5] |  |
|  | P value | 0.91 | 0.18 | 0.051 | **0.022** |  |
|  |  |  |  |  |  |  |
| **PTCy, %[95% CI]** | No PTCy | 27.1%[23.1-31.3] | 14.2%[11.2-17.6] | 40.2%[35.2-45.1] | 17.2%[13.5-21.3] |  |
|  | PTCy | 21.9%[14.5-30.3] | 8.6%[4.2-14.9] | 38.2%[27.8-48.6] | 18.2%[10.7-27.4] |  |
|  | P value | 0.33 | 0.15 | 0.81 | 0.32 |  |

**Abbreviations:** AML, acute myeloid leukemia; NRM, non-relapse mortality; LFS, leukemia-free survival; OS, overall survival; GRFS, GVHD-free, relapse-free survival; HCT, hematopoietic cell transplantation; MSD, matched sibling donor; MUD, matched unrelated donor; Haplo, haploidentical donor; 95% CI, 95% confidence interval; CK, complex karyotype, MK, monosomal karyotype; MAC, myeloablative conditioning; RIC, reduced intensity conditioning; CMV, cytomegalovirus; TCD, T-cell depletion; PTCy, post-transplant cyclophosphamide; GVHD, graft-versus-host disease; aGVHD, acute GVHD; cGVHD, chronic GVHD; ext cGVHD, extensive cGVHD

**Supplementary Table S6. Causes of death in KMT2Ar patients receiving allo-HCT**

|  | **Overall (n=237)** | **MSD**  **(n=86)** | **MUD 10/10 (n=109)** | **Haplo**  **(n=42)** |
| --- | --- | --- | --- | --- |
| **Original disease** | 136 (58.4%) | 60 (70.6%) | 60 (56.1%) | 16 (39%) |
| **GVHD** | 39 (16.7%) | 7 (8.2%) | 25 (23.4%) | 7 (17.1%) |
| **Infection** | 34 (14.6%) | 8 (9.4%) | 14 (13.1%) | 12 (29.3%) |
| **Hemorrhage** | 4 (1.7%) | 1 (1.2%) | 0 (0%) | 3 (7.3%) |
| **Second malignancy** | 3 (1.3%) | 1 (1.2%) | 2 (1.9%) | 0 (0%) |
| **MOF** | 2 (0.9%) | 0 (0%) | 1 (0.9%) | 1 (2.4%) |
| **Lymphoproliferative disorder** | 1 (0.4%) | 0 (0%) | 1 (0.9%) | 0 (0%) |
| **Interstitial pneumonia** | 1 (0.4%) | 1 (1.2%) | 0 (0%) | 0 (0%) |
| **Non-HCT related** | 8 (3.4%) | 5 (5.9%) | 2 (1.9%) | 1 (2.4%) |
| **Other HCT related** | 5 (2.1%) | 2 (2.4%) | 2 (1.9%) | 1 (2.4%) |
| **missing** | 4 | 1 | 2 | 1 |

**Abbreviations:** MSD, matched sibling donor; MUD, matched unrelated donor; Haplo, haploidentical donor; GVHD, graft-versus-host disease; MOF, multi-organ failure; HCT, hematopoietic cell transplantation;
